# Is Tuberculin a Failure?

**Published:** 1891-09

**Authors:** Karl Von Ruck

**Affiliations:** Asheville, N. C.; Member American Climatological Association, American Public Health Association American Medical Association, State Medical Society of North Carolina, Etc.


### Southern Medical Record.

A MONHLY JOURNAL OF MEDICINE AND SURGERY.

VOL. XXI. ATLANTA, GA., SEPTEMBER, 1891. No. 9.

### Original Articles.

### IS TUBERCULIN A FAILURE?

BY KARL VON RUCK, B. S., M. D.,* ASHEVILLE, N. C.

Member American Climatological Association, American Public Health Association American Medical Association, State Medical Society of North Carolina, Etc.

In assuming the consideration of this question it may be well for me to state the reasons why I consider myself justified to offer an opinion.

First, I have for a number of years devoted much of, and in later years, my entire attention to the study and cure of pulmonary tuberculosis, during which time I have treated and cared for nearly a thousand such patients and have labored earnestly and diligently for success.

Much of my clinical experience was gained under exceptionally favorable facilities by having the cases under my constant observation in my special institution for their care and treatment, and under a systematic method of frequent records of everything pertaining to the course of this disease in its progress, toward improvement and cure, or adverse termination. The patients all belonged to what may be called the better or more intelligent class of society, which largely facilitated many of the investigations.

Second. I have had as large, if not a larger experience in the use of tuberculin, than any practical observer in this coun-

*Director Sanitarium for Diseases of the Lungs and Throat, Asheville, North Carolina.

try, and believe I have seen my cases more constantly and have personally observed them more frequently than was likely to be possible with others, who applied the same method in the public institutions, charities, or in private practice.

Third. The administration of the remedy was pursued under the most painstaking observations of its effects not only upon the general condition of the patient but by frequent personal physical examinations of the chest and larynx, (even as frequently as every two hours after its application in some special cases) and by frequent daily, often hourly records of the various symptoms of the disease.

Fourth. In addition to the recorded observations of local changes and prominent symptoms careful, and scientific inquiry and records were also made and preserved of its effects upon the various excretions, especially the urine and the expectoration, they were collected for periods, and changes determined of increase or decrease, both as to quantity and the amount of solid matter ; the morphological and chemical elements were determined in our laboratory under the best methods and greatest precautions, the bacilli in the sputum estimated by Gaffky's method and the specimens from every examination are preserved for future reference.

Fifth. I have endeavored, only to see things as they really are, to keep myself free from all prejudice whatever, but if I had any prejudice to overcome, it was against the remedy, inasmuch as I was not specially enthused from my observations in Berlin, and I had theoretical reasons to distrust a method of cure under which the general health of the patient rapidly deteriorated, and which had for its object the necrosis of the tubercular tissue within the lungs in the absence of means for its removal, even before I saw its application and results in Berlin.

Sixth. I undertook the use of the remedy with the determination of avoiding the severe effects and the general reactions, because of my previous convictions that any remedy which injures the nutritive processes, can never find successful application in a wasting disease, like consumption, and because I had sufficient faith in the benefits my patients would derive from climate combined with rational treatment in my institution, to believe, that as long as I would not injure them with the remedy I could abide my time, and satisfy the clamor with minute doses, until I should know more of its action and benefits, and this led me to be the first to administer the remedy contrary to the method pursued by everybody else, who in conformity with the teachings from Berlin sought to produce that, which I as scrupulously sought to avoid.

Subsequent experience proved, that the method of administration followed by myself, for prudential reasons in the first place, avoided not only the dangers which became evident from large doses, but under its pursuit the results obtained were highly satisfactory and the method of administration advocated by me, in my earlier publications, has been accepted without material modifications by all who now continue the use of the remedy. I am therefore the only one, who has a number of months' additional experience in what may be called the new or conservative method of the administration of tuberculin.

Seventh. I am perfectly conversant with the medical literature on this subject including that of Europe and especially of Germany and am in personal private communication with the source of production of the remedy, as well as with a number of the men, who have had the remedy entrusted to them in the first place, or who have the greatest experience in its use.

However, before we answer the question, "Is tuberculin a failure?" allow me to review it in a general way and to comment upon the causes which have led to distrust its favorable influence.

With the advent of the remedy and after only a short trial of it by its discoverer in a limited number of cases, there was no clinical experience adequate to justify its general use. No one knew this better than Koch himself, and could he have had his own way, the use of the remedy would have been restricted to men who were capable of its scientific application, and of safe experimentation as to the best method of its clinical application. As it was, it came into many hands who should not then, or even now have been trusted with it, and I have heard of patients who received solutions of the remedy with directions to inject it in increasing doses every other day. In the beginning most of the patients who sought the remedy were far advanced cases and permanent cure or even lasting improvement in a considerable number could not have been expected, except from a miracle.

Such patients naturally died, and the newspapers headed their death notices, "Took Tuberculin and died," or in some such way, with as much propriety as they might have headed a notice of some other death, "Took beefsteak and died," or, "took cod liver oil and died."

Unfortunately the theory of production of a coagulation necrosis with subsequent harmless absorption under the use of large and increasing doses of Tuberculin proved erroneous and the coagulation necrosis did not occur at all, the effect being either in favorable instances a connective tissue formation, or it turned out, that the necrosis was the ordinary form attended with destructive suppuration and its dangers.

Nevertheless, many experimenters who used the remedy in what we all now consider too large and too rapidly increasing doses, saw and reported improvement and apparent cures, some of them most remarkable, and in unmistakable relation to the use of the remedy, while in other cases a rapid increase of symptoms and decline of the patient stood in presumable relation to the effects of the treatment with large doses of tuberculin.

The reason for this apparent duality of action, was first sought in the selection of cases, and the unfavorable results were attributed to the use of the remedy in *unsuitable nnd advanced stages of the disease*, although as early as January the evidences in the direction of *overdosage* were accumulating and it was particularly Professor Virchow who called attention to these conditions and gave warning of the dangers attending the then established use and method of administration.

Virchow was, however, supposed to be actuated by jealousy and to be determined to lead an opposition to the treatment, and friends of Koch and his method endeavored to answer his criticisms, not by a reconsideration of the method of its application, but by adducing clinical evidence to the good effect of the remedy by the exhibition of many cases greatly benefited and apparently cured under its use.

Thus in a session of the Society of the Charity Physicians, Dr. Stricker showed at one time twelve cases cured of pulmonary tuberculosis and reported many others greatly improved, and tending toward a recovery under the use of tuberculin, his method had, however, been slower, and from five to six months had been consumed in reaching the larger doses. He challenged the members of the Society successfully, to the production of even one single case or reference to one in the past, who at that season of the year and in the city of Berlin, had made a similar improvement and recovery under any other method of treatment, stating his willingness in that case, to attribute the surprising results he was able to demonstrate in the twelve cases, to chance and accident.

Dr. Stricker's material was specially favorable, being all soldiers, who not so very long before must have passed muster for admission in the German army and in whom the disease was early diagnosed, and the comparatively large doses were borne without injury.

In the meanwhile, good, indifferent, and bad terminations from the treatment were reported from the different public and private institutions in Germany, and the *Deutche Medizinische Wochenschrift* devoted and still devotes a considerable portion of its weekly pages to these publications, to which I must refer for particulars, the reports on the whole showing that the use of the remedy was frequently attended by brilliant results, but also by unexpected reverses, even in cases which had made considerable and unmistakable improvement, often after exhibition of increasing and very large doses, or relapses occured after the treatment was intermitted or discontinued at too early a period. These reports are however growing in proportion, as the doses are reduced and fever reactions avoided.

Most of the results obtained and reported up to a few months ago, and in some instances even to the present time, were obtained under the original mode of administration, *with purposely iudnced general and febrile reactions*, and this both in Europe and this country, and no distinct public utterances against the production of these constitutional febrile reactions or large doses were made in Germany until quite recently, although Dr. Paul Guttman and Professor Ehrlich reported in

March, their trials with minute beginning doses of 1-20th of a milligram and very gradual increase, with better results.

In this country I first called attention to these dangers in publications as early as January and again in February and March and have not only had the satisfaction of having started my use of the remedy with the object of their avoidance, and noting subsequently the acknowledgement by all experimenters that the rapidly increasing doses with severe reactions were a mistake, but also the satisfaction of having in over 2,000 injections seen no detrimental results.

With very few exceptions, in the first few weeks of my experience, I have given this remedy without even the slightest discomfort to my patients and there is no longer any use of distrusting the remedy on account of its dangers which are excluded by a knowledge of proper dosage and proper selection of cases. We might on the same principle object to any other alkaloidal remedy and other poisons, overdoses from many of which have been followed by disaster.

Since the more recent general adoption of a more conservative method in administration, the reports from Europe have been free from observations of complicating pneumonias and general infection, and the expressions of confidence in the utility of the remedy are becoming stronger and abiding from reliable sources.

It will, nevertheless, happen in the future, as in the past, that some patients who receive injections of tuberculin will fail to recover and will eventually die from their disease. As long as disease has existed and as long as people will die of it, just so long will it be possible to show that remedies which have been applied in our efforts to avert a fatal result, or to prolong life, have been unavailing in such cases. It does not, on that account, follow that the remedies which failed us under such circumstances, are without value or have killed the patient; if no remedies could be applied in disease, which have ever failed to avert a fatal result, then there is no remedy in existence which we can use in the treatment of disease.

To the apparent recoveries and improvements which I reported in my paper before the American Medical Association in Washington on May 7th,[1] I can now add at least three other successful, and quite a number of greatly improved cases, and can state that the good results in the reported apparently recoved and greatly improved cases continue, and with them no relapses have as yet occurred.

When reporting these cases I stated explicitly that I was unable to say, just how much of the good results were attributable to the tuberculin, inasmuch as I had used other means also, and I desired more particularly to show that the remedy was given without incurring the dangers which others had pointed out, and only expressed my belief that the remedy had been an aid in my hands free from danger and disagreeable effects of any kind, at any rate my results show that all of the twenty-five cases, some of which barely justified even the hope of improvement, have improved or have apparently recovered with the exception of two or three who were very far advanced and in these, the doses given were very small indeed, and in nowise produced disagreeable symptoms or aggravation of the disease.

In this country the earliest report of cases by Dr. Jacoib, to the latest by Dr. Dennison, of Denver, Colorado, are certainly encouraging when we consider that the administration of the remedy was with few exceptions upon the method first advised in Berlin, that many cases were in the last stages, or for other reasons were unsuitable, that the treatment was, with few exceptions carried out in localities with a bad climate, and at a time when at best, it was an experiment in every case.

I have elsewhere reviewed the adverse report of forty-three cases by Dr. N. Senn, in which improvement in ten, and apparent recoveries in two cases only, was shown to have attended the use of the remedy, and many cases grew worse under its use. Apart from the incomplete data furnished with this report as to the condition of the patients upon admission and upon their discharge, I have pointed out, that in at least thirteen cases the *large doses* given are probably the cause for the more rapid deterioration of the patients' condition, that sixteen other cases were hopelessly advanced or entire-

1Therapeutic Gazette, June 15th, '91.

ly unsuitable for the treatment, and that in seven others the treatment was given for such a short time (about two weeks) that it was unreasonable to expect any result. Of all these forty-three cases there were barely twelve which should have been accepted as patients for this method of treatment and in these, with few exceptions, the dosage was excessive, as it naturally had to be, the experimenter giving it to the production of general and febrile reactions.

How could, then under such circumstunces, the whole material be submitted as evidence to the disadvantage of the method? And still with all that, after an apparently very short time, from a few weeks to sixteen weeks, improvement in *ten* and apparent recovery in two cases is said to have resulted, unquestionable evidence of the good effects obtained even from large doses in cases that could stand them, or where no complications were produced. Why Dr. Senn should have felt himself justified to base adverse criticisms upon these forty-three cases treated, I fail to understand.

To me, even some of the indifferent or adverse results are still remarkable, because they were not worse, and because more serious consequences did not follow such reckless use, and continuance of the treatment, when it seemed plain enough, that the patient was not improving or was growing sicker and weaker under its use, as for instance, in one case reported from Philadelphia, the patient was rapidly run up to 10) milligrams, growing worse all the time and died the day following the 100 milligram dose. In the same length of time I frequently do not reach three milligrams and in some cases to even one milligram, I still find my patients to respond, by appreciable local effects.

Let any one who is willing to be convinced at all, with or without personal practical experience in its use, calmly review the history of tuberculin, taking into consideration the great expectations entertained both by physicians and patients, remembering that every one who made use of the remedy became an independent experimenter, and that the first theory of its action as well as the application of the remedy based upon it, was erroneous, let him consider then, not only the fact that many of the patients died, or failed to improve of a disease which is proverbally unfavorable in its course and which would, in all probability have proved fatal under the usual treatment pursued, but let him also consider the real improvements and apparent recoveries which have followed its use in many instances even with excessive doses, and then let him review the history of the therapeutics of pulmonary and other forms of tuberculosis apart from tuberculin, let him take seriatim, all other remedies and examine as critically the results which after years of effort and experience, have been produced by any of them, and compare the results. If still skeptical, let him tell us of the remedy and its name which has anything like the favorable influence we have witnessed from the experimental stage of the tuberculin treatment? What combination of the most popular remedies has ever enabled any one to show apparent cures and improvements to the degree that was obtained with tuberculin in large city hospitals at the most unfavorable season of the year and in so short a time? In a recent report by Prof. Langenbuch Deutch M. D., Wodenshift, July 23rd, the results in 35 early stage cases, are 25 recoveries, 10 cases of improvement, in the same Journal of July 30th, Dr. Thamon reports 40 per cent of cures and 45 per cent improvement, most cases having been in an advanced stage. Dr. Alfred L. Loomis reports in the *Climatologist*, August number, improvement in every one of the 9 cases in the earlier stage, in the second stage, one out of two, and in the last stage four out of five cases improved.

What course of treatment, management and combination of all possible believed beneficial influences has made a record, in which the majority of patients, early or advanced, did not grow worse or ultimately died? Indeed there are none so blind as those who do not want to see, and I do not write for those; my object in writing is, to preserve the profession from an untimely, hasty judgement in the absence of personal experience, which brings the danger of throwing overboard the wheat and the chaff together, for if tuberculin is nothing more than an aid we cannot afford to have discredit brought upon it, but need it badly enough in our efforts to deal with a disease, which is so uniformly fatal, and where every addition to our resources must be gladly welcomed.

The question whether tuberculin is a failure is of vital interest to the whole of the civilized world and in answering it, the reply must depend upon what it is expected to accomplish.

"IT IS SURELY A FAILURE," if we expect by it alone, to cure tubercular disease in its advanced stages.

IT IS LIKELY TO PROVE A FAILURE in many cases, even in the earlier stages, when dependence is placed on it alone to the exclusion of other favorable influences and it with all other advantages combined, will prove a failure in a certain class of cases with a minimum of constitutional resistance or recuperative power, cases which are doomed with the moment of acquirement of the disease, and for which nothing short of prevention can be of avail.

"IT IS SURELY NOT A FAILURE," if we expect from it a certain specific action upon tubercular tissue and acting as a local stimulant, thereby favoring and increasing nutritive repair, especially not in properly selected cases, and if we employ it under such restrictions and precautions as experience has already pointed out, and will perfect in the future.

It probably will be found "NOT TO BE A FAILURE," even in certain cases of advanced disease where it appears to aid in retarding the disease and in the accomplishment of more or less lasting improvement and arrestment.

My position then on this question is, that tuberculin is in the pulmonary affections a specific aid to local reparative processes which constitute nature's most desirable cure, by encapsuling atrophy and absorption or other subsequent changes of the tubercular tissue, this being however only an arrestment, or apparent cure, until the encapsuled tissue has undergone such changes, that re-establishment and extension of active process cannot recur, during which time relapses must be prevented, by a persistent, continuous use of the remedy, and all other means at our command.

In this connection I desire again to express my conviction that every dose of tuberculin which produces fever and other constitutional symptoms, is an overdose and that anything more than an observable, transient local effect, no matter from how small a dose, is an indication for intermission and subsequent reduction of the dose, and that if only enough pains and care are taken, and the patients are frequently examined, all disagreeable and unpleasant symptoms can be avoided, and the remedy can be employed without incurring the least danger, whatever.

Finally as stated before, the remedy is probably only an aid, but a most important one, it can never be employed in a routine way, without incurring various dangers and cannot be depended upon to the exclusion of proper management and climate and other means which are equally important aids to the local and general nutritive processes, and to the prevention of relapses, all of which constitute, and ever will constitute the successful means for the cure of pulmonary tuberculosis, until we come in possession of a true specific, which can at once eradicate not only the tubercular disease, but also the predisposition to its acquirement, by conferring immunity against its extension and against reinfection.

